# Changes of Solitude Behaviors among College Students: A Latent Transition Analysis

**DOI:** 10.3390/bs14050385

**Published:** 2024-05-02

**Authors:** Tour Liu, Fuyu Wan, Xurong Lu

**Affiliations:** 1 Key Research Base of Humanities and Social Sciences of the Ministry of Education, Academy of Psychology and Behavior, Tianjin Normal University, Tianjin 300387, China; 2 Faculty of Psychology, Tianjin Normal University, Tianjin 300387, China; 3 Tianjin Social Science Laboratory of Students’ Mental Development and Learning, Tianjin Normal University, Tianjin 300387, China; 4 School of Psychology, Shaanxi Normal University, Xi’an 710062, China

**Keywords:** positive solitude, eccentricity, social avoidance, loneliness, latent transition analysis

## Abstract

Solitude behaviors encompass four types: positive solitude, eccentricity, social avoidance, and loneliness. These four types of solitude behaviors are not entirely independent but can co-occur within individuals. Thus, the purpose of this study was to explore latent classes of solitude behaviors, their developmental patterns, and relevant influencing factors among college students. The Solitude Behavior Scale—Short Version was administered to a sample of college students. A total of 417 Chinese students completed a three-time longitudinal paper questionnaire. The data analysis was performed using Mplus 8.0 and SPSS 26.0. Harman’s single-factor test, latent class analysis (LCA), and latent transition analysis (LTA) were employed for subsequent analysis. The results revealed three classes: low solitude, moderate solitude, and high solitude, which exhibited temporal changes. Social avoidance and loneliness could facilitate transitions between high solitude and moderate solitude. Females and first-grade students exhibited higher transition probabilities than males and students not in the first grade. The incidence of moderate solitude in the not-first-grade group was significantly higher than that in the first-grade group. Finally, this study offers new insights into the dynamics of solitude behaviors and their association with gender and age.

## 1. Introduction

### 1.1. Conceptualization of Solitude Behaviors and Their Latent Classes

In recent years, the phenomenon of “being or living alone” among college students has become increasingly common, and it often leads to issues in interpersonal relationships, mental health, and physical health. Some students withdraw from social interactions due to academic pressure, environmental changes, lack of social connections, or personalities [1]. These students often exhibit higher levels of problematic mobile phone use, health issues, etc. [2,3]. Conversely, some students actively choose solitude for emotional regulation, reflection, introspection, and creativity [4]. These students typically exhibit greater life satisfaction, relaxation, and less loneliness [5]. Previous research has explored the underlying structure of solitude behaviors according to the self-determined theory, delineating solitude behaviors triggered by four distinct motives [6,7]. However, due to the complexity of intrinsic motivations, different solitude behaviors may occur simultaneously in an individual. Therefore, person-oriented analysis (e.g., latent class analysis) should be considered to explore the distinct subgroups of college students’ solitude behaviors. It is helpful for researchers to better understand the current status of solitude behaviors among college students and to develop corresponding targeted intervention measures.

What is solitude? Winnicott was among the first to acknowledge solitude, considering it as a form of capability and a crucial indicator of emotional development [8]. Goffman believed that solitude is physical separation from others [9]. Larson pointed out that solitude entails not only physical separation from others but also detachment from dependency and companionship [10]. Burger proposed that solitude is not only physical separation from others but also the absence of social interaction [11]. Subsequently, an increasing number of researchers have shifted their focus toward the psychological experience of solitude rather than solely examining it as a standalone phenomenon [12]. Long and Averill suggested that the paradigm of solitude behavior involves disengagement from immediate social demands [4]. Chen et al. pointed out that, besides having clear external behavioral characteristics, solitude is also an intrinsic personality trait [6].

For a long time, there have been two contradictory views about the significance of solitude behaviors. Some researchers considered solitude negatively, associating it with loneliness and anxiety [13]. Solitude has traditionally been regarded as a long-term criterion for intervention, often conflating with various psychological experiences such as social rejection, withdrawal, isolation, shyness, and loneliness [14]. Young adolescents often hold negative views toward solitude and find solitude aversive [15]. Nonetheless, some research has also supported the benefits of solitude on wellbeing [7,10]. Erickson proposed that resolving identity and role confusion was a significant challenge for adolescent children [16]. Long et al. asserted that solitude could afford individuals more positive experiences, including relaxation, self-reflection, creative pursuits, and emotional regulation [12]. A study found that high-arousal positive affect could persist in a solitude condition, and individuals experienced relaxation and reduced stress when actively choosing solitude [17]. Nicol applied self-determined theory to explain solitude behavior, dividing it into self-determined and non-self-determined solitude [7]. Building on Nicol’s work, Chen and her colleagues categorized solitude behaviors into positive solitude, eccentricity, social avoidance, and loneliness [6]. Positive solitude and eccentricity are driven by self-determined motivation. Positive solitude is associated with positive emotional experiences, while eccentricity is associated with negative emotional experiences [18,19]. Social avoidance and loneliness arise from non-self-determined motivation and are both associated with negative emotional experiences [20,21]. Previous research indicated that while positive solitude was associated with creativity, high self-esteem, and wellbeing, eccentricity was a significant characteristic of schizophrenia, and social avoidance and loneliness were related to psychological health issues [6,22,23].

Due to the complexity of intrinsic motivation, voluntary and involuntary solitude behaviors could occur simultaneously [24]. For example, loneliness and social avoidance are frequently discussed together [25]. Weinstein et al. discovered that spending an amount of time in involuntary solitude could lead individuals to feel more loneliness and less satisfaction [26]. Coplan et al. employed latent profile analysis to investigate the subgroups of solitude activities in adolescents and identified six classes: sociable, unsociable, balanced, alonely, socially avoidant, and shy-withdrawn [27]. Borg et al. examined the differential patterns of solitude and sociability characteristics, and their results identified four distinct classes, namely, non-solitude-social group, moderate group, affinity for solitude-sociable group, and preference for solitude-non-sociable group [28]. In Borg’s study, the solitude characteristics encompassed enjoyment, motivations, preference, and frequency [28]. This study looked into the more intricate aspects and components of solitude behaviors, using positive solitude, eccentricity, social avoidance, and loneliness as characteristics. This detailed approach offers a new way of looking at the topic and provides an updated insight that can guide future research in this field.

### 1.2. Influence of Gender and Age on Solitude

Individual differences in solitude behaviors, particularly concerning gender and age, exist [29,30]. Some evidence suggests that females exhibit higher levels of loneliness and social anxiety compared to males [31,32,33]. However, some studies have shown that males are more likely to report loneliness and adopt social avoidance strategies than females [34,35]. There are also some studies on gender differences in the transition of solitude behaviors. For example, Grills-Taquechel et al. conducted a two-year longitudinal study on sixth-grade students and observed a significant reduction in social anxiety among boys [36]. Social avoidance is frequently employed as a strategy to alleviate social anxiety [37]. Cohen-Mansfield et al. and Lin et al. both reported that older women are more likely to experience a longitudinal transition into loneliness [38,39]. However, Barreto et al. observed that, with increasing age, loneliness decreases for both men and women [34].

Solitude provides space for individuals at different stages of life (such as adolescence, early adulthood, and college life) to alleviate pressure and establish their self-identity [40,41]. A study indicated a high correlation between adolescents’ solitude and loneliness [42]. Younger people significantly report more loneliness than older people [34,43]. However, some other evidence suggested a U-shaped curve, wherein both younger and older people experience higher loneliness compared to middle-aged individuals [44,45,46]. In addition, research has shown that, over time, individuals in early adolescence start to become more willing to spend time in solitude and experience less aversion to solitude [47,48]. Based on the above, gender and age can influence an individual’s solitude and its transitions. Due to the inconsistency of previous results, these effects remain to be explored further.

### 1.3. Transitions of Solitude Behaviors

Over time, different solitude behaviors can transform into each other. Long and Averill proposed that solitude experiences are related to self-transformation and the reconstitution of cognitive structures [4]. During solitude, individuals are more likely to enter an imaginative world and engage in self-examination and reconceptualization, which may promote transitions in solitude behaviors. Solitude can help individuals reduce stress and negative emotions, but it can also exacerbate solitude severity when individuals retreat from unpleasant social occasions [10,11]. Frequent involuntary solitude may lead to social disengagement, withdrawal, and an increase in time spent alone [49]. Additionally, some individuals may enjoy solitude and have positive experiences when they recognize the benefits and, thus, mitigate negative impacts [50]. In summary, individuals’ solitude behaviors can change over time. Therefore, it is essential to explore the potential transition mechanisms of solitude behaviors.

Latent class analysis (LCA) and latent transition analysis (LTA) are valuable statistical methods for investigating the occurrence mechanism and patterns of transformation. LCA primarily addresses categorical variables, overcoming the limitation that factor analysis is restricted to continuous latent variables. Building on this, LTA additionally offers the benefit that longitudinal analysis can elucidate development trends for categorical latent variables. For example, Petersen et al. investigated the mental health status of a group of 8–9-year-old children over two years, employing LCA to analyze their mental health classes and utilizing LTA to reveal their mental health transition patterns upon entering early adolescence and their related factors [51]. Ramain et al. tracked the mania and depression dimensions of first-episode psychosis patients over three years, using LTA analysis to study classes with different emotional characteristics and their transformations over time [52]. Additionally, both LCA and LTA are methods targeted for individual analysis. LCA is employed to categorize individuals into different classes based on an individual-centered perspective [53]. Meanwhile, LTA is an individual-centered analytical method used in longitudinal data, revealing periodic change patterns and influential factors based on transition probabilities [54].

Therefore, three hypotheses are proposed:

**H1.**  *A latent class model with varying severities of solitude behaviors will exist.*

**H2.**  *The latent classes of solitude will change over time.*

**H3.**  *Gender and age* *may influence the transition probabilities.*

## 2. Methods

### 2.1. Participants

Participants were recruited to complete a longitudinal survey using a paper–pencil questionnaire. All participants were college students in Tianjin, China. All participants provided written informed consent to participate in this research and completed the questionnaire. During the initial wave in October 2020, 616 volunteers completed the survey. Subsequently, they completed the same questionnaire again after two weeks, and this process was repeated three times. The participants were required to provide a personal identification code to match their responses at each time, ensuring confidentiality and that their responses would not affect their academic performance. Eventually, 417 participants who completed all three questionnaire waves were included in the analysis, comprising 343 females and 70 males (gender codes were missing for four participants; Mean age = 19.54, SD = 1.95). Following preliminary analysis in SPSS, no outliers were detected.

### 2.2. Measures

#### Solitude Behavior Scale—Short Version (SBS-S)

We utilized the short version of the original Solitude Behavior Scale to assess the participants’ solitude behaviors, with scale items listed in Table A1. The SBS-S contained 16 items and four subscales, which were Positive Solitude, Eccentricity, Social Avoidance, and Loneliness, respectively [6,55]. Each item was scored on a five-point scale (1–5 representing “strongly disagree”, “disagree”, “neither agree nor disagree”, “agree”, and “strongly agree”). The Cronbach’s alpha coefficients of the subscales were all above 0.75, and the retest reliability at a two-month interval was above 0.60. The results of the confirmatory factor analysis supported a four-factor model (*χ*^2^/*df* = 4.74, *TLI* = 0.92, *CFI* = 0.94, *SRMR* = 0.05, *RMSEA* = 0.06). Therefore, the SBS-S demonstrated good reliability and validity, making it suitable for evaluating individuals’ solitude behaviors.

### 2.3. Statistics

Latent class analysis (LCA) and latent transition analysis (LTA) were employed in the data analysis. Initially, the 16 items of the SBS-S were utilized as observed indicators to establish a latent class model. Subsequently, the results of the latent class analysis were used to fit the latent transition model across the three measurement time points. Thirdly, the influence of gender and age on the latent transition probabilities was explored. Descriptive statistics and Harman’s single-factor method were performed using SPSS 26.0. The latent class analysis and the latent transition analysis were performed using Mplus 8.0.

## 3. Results

### 3.1. Testing of Common Method Bias

Harman’s single-factor method was utilized to assess common method bias. The data from all measurement items at the three time points underwent principal component analysis without rotation, aiming for the interpretation rate of the first common factor to fall below the critical standard values of 50% or 40% [56,57]. The findings revealed the presence of four common factors with eigenvalues exceeding 1 at T1, T2, and T3. The proportions of variances explained by the first common factor at these time points were 26.47%, 30.81%, and 31.39%, respectively. Importantly, the variance interpretation rates of the first common factor at all the three time points remained below the critical threshold of 40%, indicating that there were no significant common method biases.

### 3.2. Results of Latent Class Analysis

#### 3.2.1. Results of Latent Class Analysis

To find the best-fitting model of solitude behavior at the three time points, a total of fourteen LCA models (one-, two-, three-, and four-class models at T1; one-, two-, three-, four-, and five-class models at T2; one-, two-, three-, four-, and five-class models at T3) were computed. The indexes of these latent classes model are presented in Table 1, including AIC, BIC, Entropy, and Lo–Mendell–Rubin likelihood ratio test (LMR-LRT) [58,59]. Lower AIC and BIC values, along with higher entropy, were indicative of a better model fit. And significant results from the LMR-LRT (*p* < 0.05) suggested that it was better to accept the *k* group classification than *k* − 1 group classification. Based on Table 1, the three-class model exhibited the lowest BIC values at T1, while for T2 and T3, the four-class models displayed the lowest BIC values. However, the differences in BIC values between the three-class and four-class models were very close. Furthermore, the LMR-LRT results indicated a significant enhancement in model fit when transitioning from the two-class model to the three-class model (*p* = 0.02, *p* = 0.01). However, there was no significant improvement in model fit observed when progressing from the three-class models to the four-class models (*p* = 0.75, *p* = 0.87). Hence, the three-class models were preferred for both T2 and T3. Finally, the three-class models were suitable for analyzing measurement data across all the three time points and could be employed for subsequent latent transition analysis.

#### 3.2.2. Portraying and Naming Latent Classes According to Response Probability

**Figure 1:**
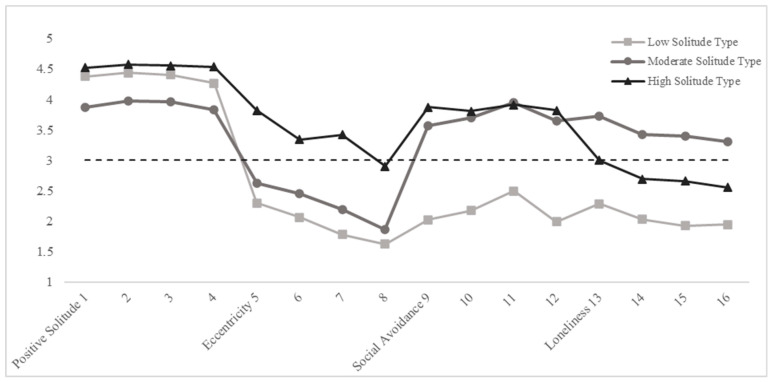
The response probabilities profile of the three latent classes. Note: The black dotted line indicates that the mean value of each item was equal to 3. When the mark of the solitude-type line is higher than the black dotted line, it means that individuals are more likely to choose high scores.

After LCA, the average scores of each participant on each item were obtained separately, and the profile of the response score was drawn (see Figure 1). The x-axis of Figure 1 corresponded to the 16 items of SBS-S, with items 1–4 reflecting positive solitude, items 5–8 reflecting eccentricity, items 9–12 reflecting social avoidance, and items 13–16 reflecting loneliness. The y-axis of Figure 1 represented the average score of the classes on each item, and it was possible to choose “strongly agree” when the value was greater than 3.

According to Figure 1, the three latent classes were named “low solitude”, “moderate solitude”, and “high solitude”, respectively. Individuals with low solitude demonstrated the lowest level of solitude across the entire scale, excelling primarily in the positive solitude dimension, while performing poorly in the other dimensions. Initially stable in number, this group experienced a slight decrease over time, with proportions of 0.33 at T1, 0.33 at T2, and 0.32 at T3. Individuals with moderate solitude exhibited a moderate level of solitude overall. They performed a little better in positive solitude but performed poorly in eccentricity, while scoring slightly higher in social avoidance and loneliness. Initially decreasing in number, this group later experienced an increase, with proportions of 0.39 at T1, 0.38 at T2, and 0.40 at T3. Individuals with high solitude displayed the highest level of solitude across the entire scale. They scored the highest in positive solitude and also scored higher in eccentricity and social avoidance. Initially increasing in number, this group later decreased, with proportions of 0.28 at T1, 0.30 at T2, and 0.28 at T3.

### 3.3. Results of Latent Transition Analysis

Table 2 and Table 3 display the transformation matrices of the latent classes. The diagonals of the matrices show the probabilities of remaining in the original classes. According to Table 2, individuals classified as having low solitude, moderate solitude, and high solitude had probabilities of 0.88, 0.79, and 0.80 respectively, of remaining in their original classes from T1 to T2. There was a 0.13 probability of transitioning from moderate solitude to high solitude. And there also was a 0.16 probability of transitioning from high solitude to moderate solitude.

According to Table 3, the three classes had probabilities of 0.90, 0.96, and 0.87, of remaining in their original classes from T2 to T3. The transition probabilities were quite low, ranging from 0.01 to 0.08.

**Figure 2:**
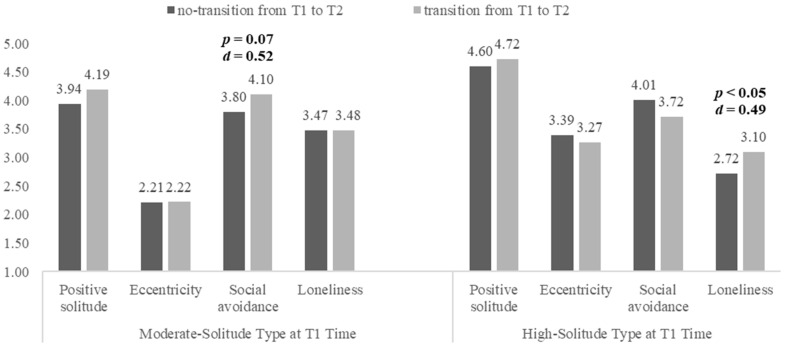
Difference testing between transformed and non-transformed individuals.

To investigate the factors influencing the transition between moderate solitude and high solitude, individuals with class numbers 2 2, 2 3, 3 3, and 3 2 at time T1 and T2 were selected. Specifically, 2 2 denotes individuals exhibiting moderate solitude at both T1 and T2. 2 3 represents individuals with moderate solitude at T1 and high solitude at T2. 3 2 indicates individuals with high solitude at T1 and moderate solitude at T2. Lastly, 3 3 refers to individuals displaying high solitude at both T1 and T2. The independent samples *t*-test was performed, and the results are presented in a bar plot (see Figure 2). The results show the differences in the scores for four solitude behaviors between the individuals who experienced transitions and those who did not during the T1 to T2 period. As illustrated in Figure 2, individuals who transitioned from moderate to high solitude at T2 scored notably higher in social avoidance compared to those who remained unchanged, although the difference was marginally significant (*p* = 0.07, Cohen’s *d* = 0.52). Subsequently, a bootstrap analysis was conducted (with 1000 random samplings), indicating that the *t* values of the 95% confidence interval were all greater than zero (*t* = 1.80, 95% CI = [0.14, 2.19], *p* < 0.05). Furthermore, individuals who shifted from high solitude at T1 to moderate solitude at T2 exhibited significantly higher scores in the loneliness subscale compared to those who maintained their original status (*p* < 0.05, Cohen’s *d* = 0.49).

### 3.4. Effect of Gender and Age on Transition Probability

#### 3.4.1. Effect of Gender on Latent Status and Transition

The odds ratio (OR) reflects the likelihood of latent classes occurring at T1 and transitions influenced by covariates. Table 4 presents results with gender serving as the covariate (where female was coded as *X* = 0, male as *X* = 1). At T1, low solitude served as the reference class, and in the transition matrix, the diagonal probabilities were used as a reference. An odds ratio greater than 1 indicates that males possess higher incidence or higher transition rates. Compared to low solitude at T1, males showed a lower occurrence for moderate solitude and high solitude. From T1 to T2, males exhibited significantly lower transition rates from moderate solitude to high solitude compared to females (OR = 0.00 ***). Similarly, from T2 to T3, the transition rates for males from moderate solitude to high solitude and from high solitude to low and moderate solitude were also significantly lower than those for females (OR = 0.00 ***).

#### 3.4.2. Effect of Age on Latent Status and Transition

Table 5 shows the results with age serving as the covariate (the low-age group was *X* = 0; the high-age group was *X* = 1). At T1, low solitude served as the reference, and in the transition matrix, the diagonal probabilities were used as a reference. An odds ratio greater than 1 indicates that the not-first-grade group possesses higher incidence or higher transition rates. The not-first-grade group had a higher occurrence in moderate solitude and high solitude at T1. From T2 to T3, the first-grade group exhibited a higher transition probability from moderate solitude to high solitude (OR = 0.00 ***).

## 4. Discussion

This study employed LCA to accurately identify heterogeneous groups/classes within college students, based on their responses to the Solitude Behavior Scale. The LTA was utilized to explore transition patterns within these heterogenous groups/classes over time and further examined the role of influencing factors in these classes and their transitions. It broadens the theoretical framework on solitude behaviors and provides new perspectives and in-depth insights into previous research on the solitude behaviors of college students.

After conducting the LCA, three latent classes were identified and labeled as low solitude, moderate solitude, and high solitude, respectively. As depicted in the response patterns profile (see Figure 1), individuals with low solitude scored higher only in the positive solitude dimension. Hence, it indicates that individuals of this class seek solitude positively. Nguyen et al. also found that positive solitude time could make people relax more and reduce stress [17]. In the moderate-solitude class, individuals scored the lowest in the positive solitude dimension compared to the other two classes, while demonstrating relatively higher scores in social avoidance and loneliness. This shows a propensity for passive solitude within this class, potentially leading to significant risks such as social anxiety symptoms, peer problems, and depressive problems [60]. In the high-solitude class, individuals scored the highest in positive solitude, eccentricity, and social avoidance. The intrinsic motivations within this class were multifaceted, comprising both self-determined motivations and non-self-determined motivations. Positive solitude and eccentricity were primarily driven by active intrinsic motivations, whereas social avoidance represented a passive withdrawal behavior aimed at alleviating anxiety [6,7]. A high severity of solitude had a negative impact on individuals. Chen et al. found that eccentricity was a characteristic of patients with schizophrenia [6]. Burger pointed out that social avoidance could be recognized as a characteristic of certain psychological disorders [11]. Therefore, low solitude reflected a group of individuals characterized by positive qualities, and moderate solitude reflected a group of individuals who withdraw passively and experience loneliness, while high solitude reflected a group of individuals who voluntarily distance themselves from others but might possess negative psychological traits.

The results of the LTA indicated that transitions occurred between the three classes over time. Individuals with moderate solitude had a slight probability of transitioning to high solitude, while individuals with high solitude had a slight probability of transitioning to moderate solitude. Notably, loneliness primarily influenced the transition from high solitude to moderate solitude, whereas social avoidance played a key role in the transition from moderate solitude to high solitude. Additionally, from T2 to T3, the numbers within the three classes remained stable. This might be attributed to the fact that this study was conducted at the onset of university. In order to adapt to the new environment, the solitude classes of individuals may not have been stable initially and may have become more stable later. According to interpersonal and attachment theory, individuals (especially college students) pursue interpersonal interaction [61,62]. Due to the difficult balance between social interaction and solitude, college students are prone to transitioning between two negative solitude classes. Wang et al. highlighted that involuntary solitude (e.g., rejection by peer) reflected individuals’ psychological distress and social difficulties, which could lead to voluntary solitude [15]. Although solitude or alone time helps people relieve negative emotions and stress, it may also perpetuate a vicious cycle if people retreat from social occasions passively [10,11]. Thus, social avoidance might play a pivotal role in exacerbating the severity of solitude. Loneliness refers to the perception of deficiency one feels when one’s relationship networks are less satisfying than one desire [63]. Lonely people are often driven by dual motivations—a desire to alleviate loneliness by interacting with others and a desire to shield themselves from potential rejection [64,65,66]. From the perspective of alleviating loneliness, seeking connections with others could help mitigate the extent of solitude behaviors, such as transitioning from high solitude to moderate solitude. From a self-protective standpoint, loneliness could amplify an individual’s feelings of isolation; for example, those with moderate solitude exhibited higher scores for loneliness. Spending a long time alone can cause damage to individuals. A longitudinal study by Kopala-Sibley and Klein indicated that the negative effects of solitude on individuals will become stronger as time goes by [67]. Therefore, future research should pay more attention to social avoidance and loneliness, thus providing a certain reference for the prevention of psychopathological problems.

Gender differences had an influence on the transition probabilities of the solitude classes (see Table 4). Compared to males, females were more prone to transition between moderate solitude and high solitude. This might have been because the females were more prone to social avoidance and loneliness than males. Some studies suggest that women are more prone to social anxiety than men [31,32]. And evidence also indicates that females are more likely to report loneliness than males [33]. Nevertheless, several conclusions were inconsistent. For example, some evidence suggests that males tend to employ avoidance coping styles more frequently and report experiencing greater loneliness compared to females [34,35]. In this study, the results tended to support that females exhibited higher levels of social avoidance and loneliness. Consequently, females were more likely to experience transitions between solitude classes. Changes in living environment (at the start of the academic term), perceived stress, and sensitivity toward interpersonal relationships all contributed to females exhibiting higher levels of interpersonal sensitivity than males. This could lead to voluntary or involuntary withdrawal from social interactions, which might be detrimental to their physical and mental wellbeing [68]. Therefore, future research should focus more on the solitude behaviors of female college students and delve deeper into the specific reasons underlying these transitions.

Age also influenced the transition probabilities of the solitude classes (see Table 5). The first-grade-group significantly promoted the transition of moderate solitude to high solitude from T2 to T3. The first year of college is generally a stressful and challenging life period. Most first-year college students frequently encounter feelings of loneliness and isolation [69]. Cutrona discovered that 75% of new freshman college students reported feeling lonely during their first two weeks [70]. Loneliness is frequently linked to depression [7]. First-year college students who exhibit high levels of avoidance behaviors may lack social competencies, which can contribute to feelings of loneliness, subsequent mental health issues, and academic difficulties [71]. Furthermore, the results also found that the not-first-grade group had a higher occurrence rate of solitude behaviors than the first-grade group. This could be attributed to their underdeveloped skills in positive solitude and social interactions during their first year. Therefore, if the solitude behavior patterns of first-grade college students can be identified, then ways of helping them enhance their social skills in order to build satisfactory relationships might be developed, thereby reducing loneliness and promoting successful adjustment to college life.

The findings of this study also carry some implications for future research. Firstly, it will help college individuals reduce the risk caused by negative solitude if they correctly identify their solitude types. Prolonged periods of an alone environment reinforce the negative effects of solitude [72,73]. Hence, organizing some activities can help individuals recognize the value of positive solitude and equip them with coping strategies against negative solitude [4,17,74]. Secondly, females and the first-grade group tend to transform their solitude types. Thus, there should be more attention on them because these transitions are not considered beneficial. Further consideration should be given, for example, to other individual characteristics and different developmental stages (children, adolescents, elderly, etc.), thereby providing more empirical references for establishing an integrated framework of solitude behaviors. Finally, this study used self-report measures, which can effectively assess individuals’ performance in solitude behaviors. Future research can also use more diverse approaches (e.g., intervention, neural and physiological perspectives) to examine individuals’ solitude behaviors and provide more appropriate guidance for relevant research.

## 5. Conclusions

We found three classes among individuals’ solitude behaviors; they were low solitude, moderate solitude, and high solitude, respectively.

Over time, the classes’ status remained relatively stable. Loneliness and social avoidance were identified as contributing factors to transitions between moderate solitude and high solitude.

Gender and age could influence the transition probabilities. Females and the first-grade group were more likely to transition between solitude classes.

## Figures and Tables

**Table 1:** The Indexes of model fit at three time points.

Time Points	Latent Classes	AIC	BIC	Entropy	LMR-LRT (*p*)
T1	1	18,204.22	18,462.34		
	2	17,311.75	17,832.02	0.92	0.59
	3	16,767.55	17,549.97	0.92	0.77
	4	16,545.34	17,589.91	0.93	0.77
T2	1	18,099.78	18,353.71		
	2	17,016.79	17,528.69	0.88	0.04
	3	16,396.66	17,166.52	0.94	0.02
	4	16,068.48	17,096.30	0.96	0.75
	5	15,815.32	17,101.11	0.94	0.77
T3	1	18,195.95	18,454.07		
	2	17,027.47	17,547.74	0.90	<0.001
	3	16,378.88	17,161.30	0.94	0.01
	4	16,021.05	17,065.62	0.94	0.87
	5	15,799.11	17,105.83	0.94	0.83

**Table 2:** Transition matrix of latent classes from T1 to T2.

T1	T2		
	Low Solitude	Moderate Solitude	High Solitude
Low Solitude	0.88	0.06	0.06
Moderate Solitude	0.08	0.79	0.13
High Solitude	0.04	0.16	0.80

Note: The rows represent the latent classes in T1 time, and the columns represent the latent classes in T2 time.

**Table 3:** Transition matrix of latent classes from T2 to T3.

T2	T3		
	Low Solitude	Moderate Solitude	High Solitude
Low Solitude	0.90	0.08	0.03
Moderate Solitude	0.03	0.96	0.01
High Solitude	0.06	0.06	0.87

Note: The rows represent the latent classes in T2 time, and the columns represented the latent classes in T3 time.

**Table 4:** The odds ratio of incidence and transition based on gender.

	Low Solitude	Moderate Solitude	High Solitude
OR at T1	—	0.94	0.91
OR of transition fromT1 to T2 ^a^			
Low Solitude	—	3.27	0.00
Moderate Solitude	1.93	—	0.00 ***
High Solitude	0.27	1.61	—
OR of transition from T2 to T3 ^a^			
Low Solitude	—	0.68	2.77
Moderate Solitude	2.14	—	0.00 ***
High Solitude	0.00 ***	0.00 ***	—

Note: ^a^ The rows represent the latent classes of the previous time point, and the columns represent the latent classes of the next time point; *** *p* < 0.001. The odds ratio can be calculated using the following formula: $odds\;ratio=e^{\beta }=\frac{P\left(C_{t+1}|X=1\right)/P(C_{t}|X=1)}{P\left(C_{t+1}|X=0\right)/P(C_{t}|X=0)}$, where *X* = 1 represents male, *X* = 0 represents female, and $P(C_{t}|X=1)$ represents the probability of males belonging to the $C_{t}$ status.

**Table 5:** The odds ratio of incidence and transition based on age.

	Low Solitude	Moderate Solitude	High Solitude
OR at T1	—	1.71 *	1.55
OR of transition fromT1 to T2 ^a^			
Low Solitude	—	4.64	0.00
Moderate Solitude	0.86	—	1.30
High Solitude	0.40	0.37	—
OR of transition from T2 to T3 ^a^			
Low Solitude	—	3.97	0.95
Moderate Solitude	2.35	—	0.00 ***
High Solitude	0.00	1.20	—

Note: ^a^ The rows represent the latent classes of the previous time point, and the columns represent the latent classes of the next time point; * *p* < 0.05, *** *p* < 0.001. The odds ratio can be calculated using the following formula: $odds\;ratio=e^{\beta }=\frac{P\left(C_{t+1}|X=1\right)/P(C_{t}|X=1)}{P\left(C_{t+1}|X=0\right)/P(C_{t}|X=0)}$, where *X* = 1 represents the not-first-grade group, *X* = 0 represents the first-grade group, and $P(C_{t}|X=1)$ represents the probability of the not-first-grade group belonging to the $C_{t}$ status.

## Data Availability

The original data are available on reasonable request from the corresponding author.

## Appendix A

**Table A1:** Solitude Behavior Scale—Short Version (SBS-S).

Item Number	Item
1	When I’m alone, I can engage in activities that truly interest me.
2	I think that solitude reflection can assist in one’s internal growth.
3	I can organize my thoughts better when I’m alone.
4	Sometimes I enjoy quietly reading and contemplating alone.
5	I like to be alone and have little interest in other people.
6	I feel that other people’s matters have little relevance to me.
7	I prefer to work alone rather than with other people.
8	I prefer to keep to myself and have little interest in having close friends.
9	I usually feel uncomfortable when I’m with a group of unfamiliar people.
10	I feel nervous when talking to people I’m not very familiar with.
11	I often feel like I’m not confident enough in social situations.
12	I feel uneasy in situations with many people.
13	I feel lonely when I’m alone.
14	I feel like I rely on others a bit too much.
15	I often worry about being abandoned by friends or lovers.
16	I often feel lonely when I’m alone.

Note: Items 1–4 belong to the positive solitude subscale; items 5–8 belong to the eccentricity subscale; items 9–12 belong to the social avoidance subscale; items 13–16 belong to the loneliness subscale. There are no reverse-scored items.
